# Supplementary material for: Exploiting V-Gene Bias for Rapid, High-Throughput Monoclonal Antibody Isolation from Horses
Source: Viruses. 2022 Sep 30;14(10):2172. doi: 10.3390/v14102172 (PMC9609571; doi:10.3390/v14102172)
Supplement: Supplementary file 1 [file viruses-14-02172-s001.zip › viruses-1862361-supplementary.pdf]

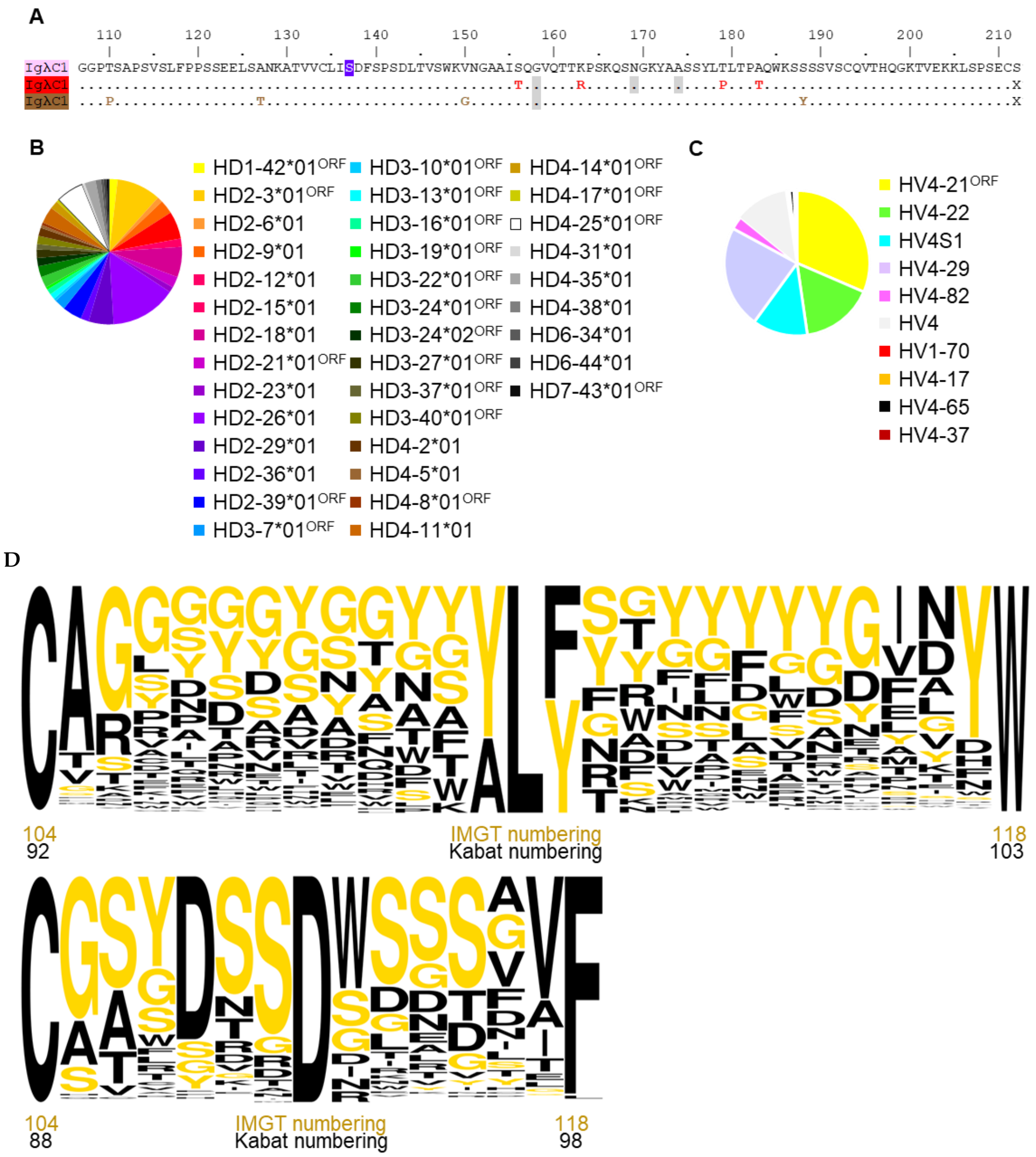

Supplementary Figure S1 (**A**) Alignment of the IgλC1 alleles identified in this study. Amino acid differences are indicated with the coloured letters, while synonymous mutations to cytosine are highlighted in grey. (**B**) IgHD pie chart from Figure 2E, with labels. (**C**) NCBI IgH*V*-gene data from Resenfeld et al., reanalysed here for comparison to the recently described IMGT® genes/alleles. Analysis showed a similar HV4-21, HV4-22, HV4S1, HV4-29, and HV4-82 distribution to our own data. The HV4 (light grey) slice includes these same genes but could not be called by IMGT® and requires manual curation, owing to the high similarity between these five HV genes. (**D**) Logograms from Figure 2H and 2I, not accounting for gaps to better show amino acids composition.
